# Novel criteria to classify ARDS severity using a machine learning approach

**DOI:** 10.1186/s13054-021-03566-w

**Published:** 2021-04-20

**Authors:** Mohammed Sayed, David Riaño, Jesús Villar

**Affiliations:** 1 Banzai Research Group On Artificial Intelligence, Department of Computer Engineering, Universitat Rovira I Virgili, Av Paisos Catalans 26, 43007 Tarragona, Spain; 2 Centro de Investigación Biomédica en Red de Enfermedades Respiratorias, Instituto de Salud Carlos III, Madrid, Spain; 3 Multidisciplinary Organ Dysfunction Evaluation Research Network, Research Unit, Hospital Universitario Dr Negrín, Barranco de la Ballena s/n, 4th Floor -South Wing, 35019 Las Palmas de Gran Canaria, Spain; 4 Keenan Research Center for Biomedical Science at the Li Ka Shing Knowledge Institute, St Michael’s Hospital, Toronto, ON Canada

**Keywords:** Intensive care units, Acute respiratory distress syndrome, Lung severity, Machine learning, Prediction models

## Abstract

**Background:**

Usually, arterial oxygenation in patients with the acute respiratory distress syndrome (ARDS) improves substantially by increasing the level of positive end-expiratory pressure (PEEP). Herein, we are proposing a novel variable [PaO_2_/(FiO_2_xPEEP) or P/FP_E_] for PEEP ≥ 5 to address Berlin’s definition gap for ARDS severity by using machine learning (ML) approaches.

**Methods:**

We examined P/FP_E_ values delimiting the boundaries of mild, moderate, and severe ARDS. We applied ML to predict ARDS severity after onset over time by comparing current Berlin PaO_2_/FiO_2_ criteria with P/FP_E_ under three different scenarios. We extracted clinical data from the first 3 ICU days after ARDS onset (*N* = 2738, 1519, and 1341 patients, respectively) from MIMIC-III database according to Berlin criteria for severity. Then, we used the multicenter database eICU (2014–2015) and extracted data from the first 3 ICU days after ARDS onset (*N* = 5153, 2981, and 2326 patients, respectively). Disease progression in each database was tracked along those 3 ICU days to assess ARDS severity. Three robust ML classification techniques were implemented using Python 3.7 (LightGBM, RF, and XGBoost) for predicting ARDS severity over time.

**Results:**

P/FP_E_ ratio outperformed PaO_2_/FiO_2_ ratio in all ML models for predicting ARDS severity after onset over time (MIMIC-III: AUC 0.711–0.788 and CORR 0.376–0.566; eICU: AUC 0.734–0.873 and CORR 0.511–0.745).

**Conclusions:**

The novel P/FP_E_ ratio to assess ARDS severity after onset over time is markedly better than current PaO_2_/FiO_2_ criteria. The use of P/FP_E_ could help to manage ARDS patients with a more precise therapeutic regimen for each ARDS category of severity.

**Supplementary Information:**

The online version contains supplementary material available at 10.1186/s13054-021-03566-w.

## Background

Acute respiratory distress syndrome (ARDS) is an acute and intense inflammatory disease process of the lungs with an associated high mortality rate of about 40% in non-COVID-19 ARDS patients [1, 2]. ARDS is a highly heterogeneous syndrome without a specific diagnostic test [3–5]. According to the LUNG-SAFE study, ARDS is unrecognized in more than half of patients at the time of fulfillment of ARDS criteria [1]. The current “Berlin definition” is under controversy [5–8]. The previous American-European Consensus Conference (AECC) [9] and the Berlin definitions are predominantly based on the value of the PaO_2_/FiO_2_ ratio at the time of ARDS onset [10].

A working definition of ARDS is essentially required for clinical trials, epidemiologic studies, and biological studies. Moreover, a definition of ARDS is required for clinicians to initiate treatments that would improve clinical outcomes [11], although stratification of ARDS—as defined by Berlin criteria—has been shown not very useful for assessing lung severity [8, 12]. The empirical PaO_2_/FiO_2_ cut-offs for “severity” of 100, 200, and 300 mmHg are arbitrary and poorly validated [13]. A recently published Reevaluation of Systemic Early Neuromuscular Blockade (ROSE) trial emphasized the variability of these PaO_2_/FiO_2_ cut-offs as the investigators did not enroll patients based on the PaO_2_/FiO_2_ at the time of ARDS onset, but based on a PaO_2_/FiO_2_ < 150 mmHg within the first 48-h after ARDS diagnosis [14, 15]. The PaO_2_/FiO_2_ ratio strongly depends on ventilator settings, including positive end-expiratory pressure (PEEP), inspiratory/expiratory time (I:E) ratio, and FiO_2_, and the requirement of a minimum PEEP of 5 cmH_2_O did not substantially improve Berlin prediction compared to AECC [13, 16]. Besides, Berlin definition does not account for the nonlinear relationship of PaO_2_ and FiO_2_ [17] and has a limited predictive accuracy in recent trials [18–21].

Assessment of severity in ARDS remains a challenge. The relation between oxygenation and prognosis in ARDS varies among published reports [20]. For example, the current mild ARDS category may not be significantly associated with 28-day mortality [22–24]. However, although stratification of severity based on Berlin criteria may be helpful to identify severe ARDS patients, it may have less significance to differentiate between mild and moderate ARDS [20]. A recent study identified two different subgroups of moderate ARDS using a 150 mmHg PaO_2_/FiO_2_ threshold and may represent a more homogeneous distribution of ARDS patients across subgroups of severity [25–27]. Whether ARDS outcome relates to severity of respiratory failure [28], a higher severity is a risk factor for prolonged mechanical ventilation [19]. Since PaO_2_/FiO_2_ does not account for PEEP in its calculation, reported PaO_2_/FiO_2_ provides a sense of ARDS severity without knowledge of applied PEEP levels.

The main goal of this study was proposing a novel variable [PaO_2_/(FiO_2_xPEEP)] or P/FP_E_ for PEEP ≥ 5 cmH_2_O that, together with corresponding thresholds, could serve as an improved criterion to assess ARDS severity. The thresholds are 60 to 40 mmHg/cmH_2_O for mild, 40 to 20 for moderate, and less than 20 for severe. This new criterion adequately addressed Berlin’s definition gap in computing ARDS severity by including PEEP in the new oxygenation ratio. Increasing the PEEP level with the same FiO_2_ yields different PaO_2_ and SpO_2_ [29]. Thus, including PEEP in calculating the degree of oxygenation severity could be better than current Berlin definition. We examined this hypothesis by applying machine learning (ML) approaches for predicting ARDS severity over time.

## Methods

### Study design and patient population

Two clinical databases were used for evaluation. Data of the first 3 ICU days (considering day 1 for representative data within the first 24 h after ARDS onset, day 2 for data within 24–48 h after onset, and day 3 for data within 48–72 h after onset) (*N* = 2738, 1519, and 1341 patients, respectively) were extracted from a single-center database MIMIC-III (MetaVision, 2008–2012) [30]. The median length of an ICU stay (LOS) of all selected ARDS patients in MIMIC-III was 11.29 days (Q1–Q3: 7.85–17.54). Similarly, data of the first 3 ICU days after ARDS onset (*N* = 5153, 2981, and 2326 patients, respectively) were extracted from a multicenter database eICU (2014–2015) [31]. The median length of an ICU LOS of all selected ARDS patients in eICU was 11.72 days (6.92–18.84). All selected patients from both databases fulfilled the Berlin criteria for ARDS and were stratified into mild, moderate or severe ARDS [6] and received mechanical ventilation (MV) for > 48 h [32, 33]. Disease progression of ARDS in each database was tracked along those 3 ICU days to assess lung severity. Patients younger than 18 years were excluded.

### Data extraction

Clinical data of ARDS patients were extracted from both databases (MIMIC-III and eICU) using Python 3.7, an interpreted, interactive, object-oriented, open-source programming language. The selection of clinical variables was based on previous studies [1, 19, 34–37].

### MIMIC-III

**Fig. 1:**
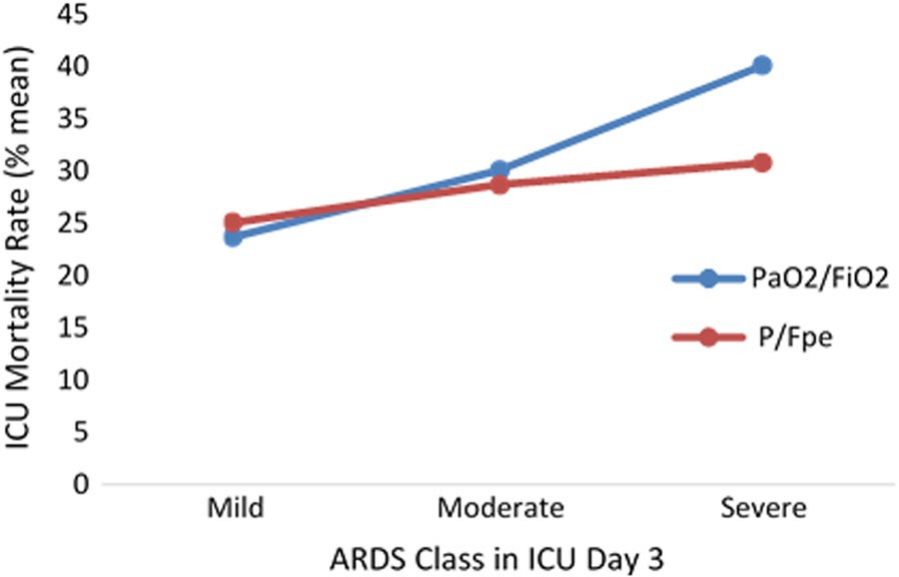
Intensive Care Unit mortality at 72 h in relation to degree of lung severity in patients with acute respiratory distress syndrome included in the MIMIC-III database, according to PaO_2_/FiO_2_ ratio and P/FP_E_ (see text for details)

MIMIC-III is a large, publicly available database including de-identified health-related data of approximately 60,000 admissions of ICU patients [30]. The input variables include baseline demographic information (age); hemodynamic parameters including mean, maximum and minimum heart rate (HR); ventilator parameters including mean, maximum and minimum respiratory rate (RR), SpO_2_, and PEEP. These predictors on the third ICU day after assessing lung severity, including their description (mean and 95% CI), are presented in Table 1, and Additional file 1: Tables S1 and S2. The main target variable was ARDS severity (where 0 = mild, 1 = moderate, and 2 = severe). ICU mortality (Fig. 1, Additional file 1: Figs. S1 and S2) and duration of MV were also obtained (Additional file 1: Table S3).

**Table 1:** Input variables and their descriptive statistics in MIMIC-III at 72-h

	Mild	Moderate	Severe	All
*A. ARDS patients*	506 (37.73%)	678 (50.56%)	157 (11.71%)	1341 (100%)
*B. Descriptive feature—means and 95% CI*				
Age	61.77 [60.37, 63.17]	60.61 [59.42, 61.79]	60.24 [57.42, 63.07]	61.01 [60.14, 61.87]
PEEP	7.41 [7.11, 7.71]	9.40 [9.06, 9.75]	11.68 [10.83, 12.52]	8.92 [8.68, 9.16]
Heart Rate_Mean	92 [90, 94]	92 [91, 94]	96 [93, 99]	93 [92, 94]
Respiratory Rate_Mean	21 [20, 21]	21 [21, 22]	22 [21, 23]	21 [21, 22]
Heart Rate_Max	114 [112, 116]	114 [112, 116]	120 [116, 124]	115 [113, 116]
Heart Rate_Min	75 [74, 77]	76 [75, 78]	78 [75, 81]	76 [75, 77]
Respiratory Rate_Max	30 [29, 31]	30 [29, 31]	32 [31, 34]	30 [30, 31]
Respiratory Rate_Min	13 [13, 14]	13 [13, 14]	13 [13, 14]	13 [13, 14]
SpO_2__Mean	97 [97, 98]	96 [96, 97]	96 [95, 96]	97 [96, 97]
SpO_2__Max	100 [100, 101]	100 [99, 100]	100 [99, 100]	100 [99, 100]
SpO_2__Min	90 [89, 90]	88 [87, 89]	85 [83, 87]	88 [88, 89]

### eICU

**Fig. 2:**
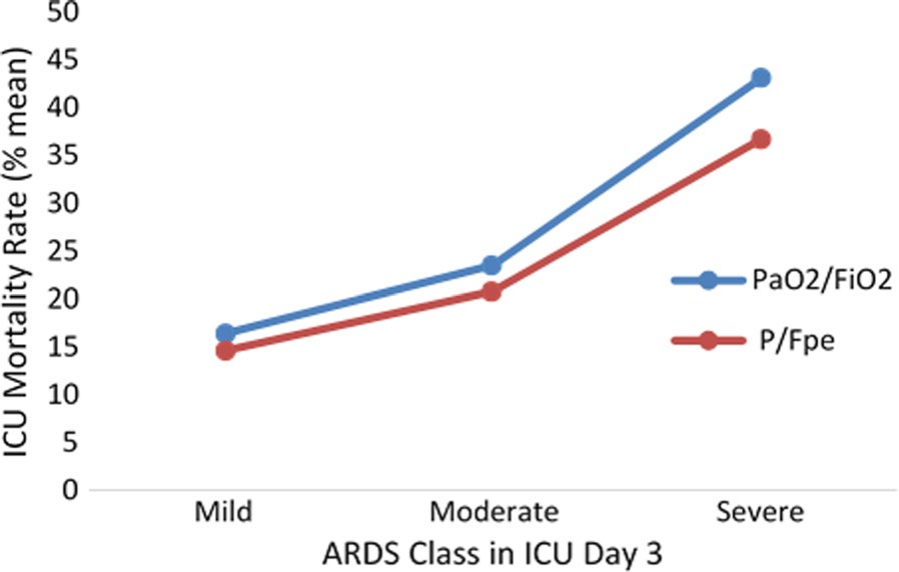
Intensive Care Unit mortality at 72 h in relation to degree of lung severity in patients with acute respiratory distress syndrome included in the eICU database, according to PaO_2_/FiO_2_ ratio and P/FP_E_ (see text for details)

eICU is a multicenter and publicly available ICU database with high level of detail in the data about more than 200,000 ICU admissions [31]. Input variables included: baseline demographic information (age); ventilator parameters including PEEP; blood gas parameters including FiO_2_, PaO_2_, and PaCO_2_ (Table 2, Additional file 1: Tables S4 and S5). The main target variable was ARDS severity (where 0 = mild, 1 = moderate, and 2 = severe). ICU mortality (Fig. 2, Additional file 1: Figs. S3 and S4) and duration of MV were also obtained (Additional file 1: Table S6).

**Table 2:** Input variables and their descriptive statistics in eICU at 72-h

	Mild	Moderate	Severe	All
*A. ARDS patients*	872 (37.49%)	1025 (44.07%)	429 (18.44%)	2326 (100%)
*B. Descriptive feature—means and 95% CI*				
Age	64.77 [63.78, 65.76]	62.73 [61.83, 63.64]	59.97 [58.67, 61.28]	62.99 [62.39, 63.59]
PEEP	5.95 [5.80, 6.09]	7.16 [6.99, 7.34]	10.09 [9.72, 10.46]	7.25 [7.12, 7.38]
FiO_2_	0.40 [0.39, 0.41]	0.50 [0.49, 0.51]	0.81 [0.79, 0.83]	0.52 [0.51, 0.53]
PaO_2_	98.89 [97.17, 100.62]	80.52 [79.25, 81.78]	74.81 [72.83, 76.79]	86.36 [85.34, 87.37]
PaCO_2_	39.93 [39.33, 40.53]	42.38 [41.72, 43.04]	44.23 [43.13, 45.33]	41.80 [41.38, 42.23]

### Experimental methods

Before starting our analysis, the thresholds of the P/FP_E_ index (with PEEP ≥ 5) were experimentally tuned. We computed the minimum and maximum P/FP_E_ values of the patients in the two databases, which were 2 and 60 mmHg/cmH_2_O, respectively. Then, several cut-offs were studied in order to determine the ones that could be more accurate in the stratification of ARDS severity. For this purpose, we tested round values (to be easily remembered by intensivists) in the range 2–60 and analyzed P/FP_E_ index of the ARDS severity groups obtained. The partition showing a better separation of the ARDS severity groups obtained was achieved in this study for the following thresholds (with PEEP ≥ 5): 60–40 for mild, 40–20 for moderate, and < 20 for severe.

Our study is based only on ML analysis and not on the conventional statistical hypothesis testing analysis. In general, ML is an exploratory process and a current application of artificial intelligence to generate predictive models. Using this technology, there is not a one-model-fits-all solution. Precisely, there is no ML method that reaches the highest accuracy for all domains, datasets, or problem types [38]. The optimal model differs from one problem to another based on the characteristics of variables and observations. Our aim was to implement ML models capable of predicting ARDS severity over time to compare the PaO_2_/FiO_2_ ratio—as mandated by the current Berlin criteria for ARDS—with the proposed new P/FP_E_ ratio according to the following three scenarios: (1) Scenario I: predicting ARDS severity in the 3^rd^ ICU day using information captured in the 1st ICU day; (2) Scenario II: predicting ARDS severity in the 3^rd^ ICU day using information captured in the 2nd ICU day; (3) Scenario III: predicting ARDS severity in the 3^rd^ ICU day using information captured in the 1st and 2nd ICU days.

We implemented three robust supervised ML algorithms using Python 3.7. The ML algorithms were Light Gradient Boosting Machine (LightGBM) [39], Random Forest (RF) [40], and eXtreme Gradient Boosting (XGBoost) [41]. Grid search was used to identify the optimal values for their input parameters. The quality of the prediction models was computed based on a tenfold cross-validation approach. AUC and CORR (correlation between the predicted and actual values of severity level) were used to assess model performance in predicting ARDS severity as a categorical prediction. To provide a meaning to the findings, we used the classification of performance suggested by Hosmer and Lemeshow [42]: “excellent” if AUC ≥ 0.9; “good” if AUC is between 0.8 and 0.9; “fair” if AUC is between 0.7 and 0.8; “poor” if AUC is between 0.6 and 0.7; and “very poor” if AUC is below 0.6. For CORR, we used the interpretation suggested by Mukaka [43] who proposed “very high” for CORR ≥ 0.9 (positive correlation) or CORR ≤ -0.9 (negative correlation); “high” if CORR is between 0.7 and 0.9 (positive) or -0.9 and -0.7 (negative); “moderate” if CORR is between 0.5 and 0.7 (positive) or -0.7 and -0.5 (negative); “low” if CORR is between 0.3 and 0.5 (positive) or -0.5 and -0.3 (negative), and “negligible” otherwise.

## Results

The findings of the three classification ML methods for the three predictive scenarios in the two databases are presented in Tables 3 and 4. Table 3 shows the quality of ML predictions for MIMIC-III, confronting the results obtained for PaO_2_/FiO_2_ (Table 3(a)) with those obtained for P/FP_E_ (Table 3(b)). Table 4 shows the same comparative results in patients from the eICU database.

**Table 3:** Quality of the third ICU day severity predictive ML models for MIMIC-III

Algorithm	AUC, mean ± SD	CORR, mean ± SD
**(a) PaO**_**2**_**/FiO**_**2**_** results**		
*Scenario I: Predicting ARDS Severity in the 3rd ICU day using the data in 1st ICU day*		
XGBoost	0.616 ± 0.039	0.190 ± 0.068
RF	0.622 ± 0.048	0.173 ± 0.089
LightGBM	0.612 ± 0.039	0.138 ± 0.084
*Scenario II: Predicting ARDS Severity in the 3rd ICU day using the data in 2nd ICU day		
XGBoost	0.621 ± 0.023	0.147 ± 0.121
*RF	0.635 ± 0.020	0.139 ± 0.094
LightGBM	0.622 ± 0.025	0.126 ± 0.120
*Scenario III: Predicting ARDS Severity in the 3rd ICU day using the data in 1st & 2nd ICU days*		
XGBoost	0.619 ± 0.030	0.150 ± 0.106
RF	0.627 ± 0.022	0.177 ± 0.108
LightGBM	0.618 ± 0.022	0.086 ± 0.101
**(b)** **P/FP**_**E**_ ** results**		
*Scenario I: Predicting ARDS Severity in the 3rd ICU day using the data in 1st ICU day*		
XGBoost	0.711 ± 0.029	0.385 ± 0.064
RF	0.712 ± 0.027	0.408 ± 0.060
LightGBM	0.716 ± 0.029	0.376 ± 0.073
*Scenario II: Predicting ARDS Severity in the 3rd ICU day using the data in 2nd ICU day		
XGBoost	0.785 ± 0.025	0.514 ± 0.053
RF	0.787 ± 0.023	0.546 ± 0.061
*LightGBM	0.788 ± 0.020	0.566 ± 0.044
*Scenario III: Predicting ARDS Severity in the 3rd ICU day using the data in 1st & 2nd ICU days*		
XGBoost	0.782 ± 0.025	0.548 ± 0.049
RF	0.780 ± 0.023	0.538 ± 0.065
LightGBM	0.785 ± 0.021	0.511 ± 0.055

^*^Identifies the optimal scenario and ML model

**Table 4:** Quality of the third ICU day severity predictive ML models for eICU

Algorithm	AUC, mean ± SD	CORR, mean ± SD
**(a) PaO**_**2**_**/FiO**_**2**_** results**		
*Scenario I: Predicting ARDS Severity in the 3rd ICU day using the data in 1st ICU day*		
XGBoost	0.712 ± 0.032	0.398 ± 0.061
RF	0.714 ± 0.030	0.393 ± 0.059
LightGBM	0.713 ± 0.028	0.373 ± 0.069
*Scenario II: Predicting ARDS Severity in the 3rd ICU day using the data in 2nd ICU day		
*XGBoost	0.863 ± 0.016	0.725 ± 0.028
RF	0.863 ± 0.016	0.700 ± 0.040
LightGBM	0.860 ± 0.014	0.714 ± 0.028
*Scenario III: Predicting ARDS Severity in the 3rd ICU day using the data in 1st & 2nd ICU days*		
XGBoost	0.860 ± 0.015	0.717 ± 0.025
RF	0.854 ± 0.017	0.693 ± 0.038
LightGBM	0.857 ± 0.014	0.713 ± 0.027
**(b) ** **P/FP**_**E**_ ** results**		
*Scenario I: Predicting ARDS Severity in the 3rd ICU day using the data in 1st ICU day*		
XGBoost	0.735 ± 0.034	0.525 ± 0.056
RF	0.735 ± 0.034	0.514 ± 0.057
LightGBM	0.734 ± 0.034	0.511 ± 0.053
*Scenario II: Predicting ARDS Severity in the 3rd ICU day using the data in 2nd ICU day		
*XGBoost	0.873 ± 0.022	0.745 ± 0.033
RF	0.868 ± 0.016	0.739 ± 0.039
LightGBM	0.869 ± 0.023	0.728 ± 0.043
*Scenario III: Predicting ARDS Severity in the 3rd ICU day using the data in 1st & 2nd ICU days*		
XGBoost	0.872 ± 0.020	0.725 ± 0.040
RF	0.860 ± 0.015	0.731 ± 0.038
LightGBM	0.871 ± 0.022	0.717 ± 0.040

For MIMIC-III, the best ML severity predictive model on the third ICU day was obtained by scenario II and by P/FP_E_ with an AUC = 0.788 and CORR = 0.566, using LightGBM algorithm. When PaO_2_/FiO_2_ is used, AUC = 0.635 and CORR = 0.19, but these performances were obtained with different algorithms. In qualitative terms, P/FP_E_ ratio improves PaO_2_/FiO_2_ ratio from “poor” to “fair” AUC, and from “negligible” to “moderate” CORR.

For the eICU database, the results were slightly better. The best ML severity predictive model was also observed for scenario II. This finding confirms that the best approach to predict ARDS severity on the third ICU day is to consider the condition of the patient in the second ICU day after ARDS onset, rather than the first ICU day or both. For eICU data, the best AUC and CORR values are 0.873 and 0.745 for P/FP_E_; and 0.863 and 0.725 for PaO_2_/FiO_2_. These results are qualified as a “good” predictive accuracy and a “high” correlation.

In general, P/FP_E_ ratio has a better behavior in the prediction of ARDS severity than PaO_2_/FiO_2_ ratio in terms of AUC and CORR. Whereas PaO_2_/FiO_2_ obtained up to 0.635 AUC and up to 0.19 CORR in MIMIC-III, the use of P/FP_E_ reached 0.788 AUC and 0.566 CORR. This represents increments of + 0.153 AUC and + 0.376 CORR and shows the advantages of using the P/FP_E_ ratio.

## Discussion

In this large study, we propose a novel variable or formula (P/FP_E_) and corresponding thresholds for classifying ARDS severity. We investigated several ML methods to generate severity predictive models in almost 8,000 patients with ARDS over time after ARDS diagnosis. Our findings confirmed that the best approach to predict ARDS severity on the third ICU day is to consider the condition of the patient in the second ICU day after ARDS onset, rather than during the first ICU day as mandated by Berlin criteria.

For the MIMIC-III database, predictive models using the P/FP_E_ ratio attained outstanding improvements in terms of AUC (15% improvement) and CORR (37.6% improvement), when compared to the previous PaO_2_/FiO_2_ models. For the eICU database, models based on P/FP_E_ also outperformed PaO_2_/FiO_2_ predictions, with 14.8% and 2% improvements of AUC and CORR, respectively. The difference in terms of the accuracy between the two databases is remarkable regarding CORR. This is due to the fact that eICU is a multicenter ICU database with high granularity data (i.e., high level of detail in the data) for over 200,000 admissions to ICUs. By contrast, MIMIC-III is a single-center ICU database for approximately 60,000 admissions of ICU patients. Therefore, in all extracted data of the three ICU days, the number of extracted patients from eICU was greater than the number of extracted patients from MIMIC-III. Consequently, this would lead to better ML results in terms of CORR for the eICU database. Overall, the novel P/FP_E_ ratio outperformed the PaO_2_/FiO_2_ ratio in all ML applied models and showed that predictions based on the patient condition in the second day after onset are better than predictions based on the first 24 h (7.2–13.8% AUC and 1.5–22% CORR improvements), followed by the predictions based on both the first and the second day conditions (0.1–0.3% AUC and 0.18–14% CORR improvements).

In contrast to our study, most recent studies developed ML approaches to predict the risk of ARDS in critically ill patients prior to ARDS onset [36, 44, 45], based on single-center databases [36, 45] and using one single ML algorithm [36, 44]. Consequently, their findings have serious limitations for the generalizability in the context of assessing the prediction of ARDS outcome.

This large study proposes a novel criterion to reclassify ARDS patients in terms of severity by using ML methods on an extensive amount of data from two large datasets of critically ill patients. The relatively good accuracy of P/FP_E_ (when compared to PaO_2_/FiO_2_) in stratifying ARDS patients could allow to overcome the major clinical drawbacks of the current Berlin definition. Also, this study is implementing ML models for predicting severity over time after ARDS onset. Critically ill patients are an ideal population for clinical database investigations using machine learning algorithms because while the data from ICUs are extensive, the value of many diagnostic and therapeutic interventions remains largely unproven [46].

ARDS is considered one of the major reasons of ICU admission, and it is associated with a high hospital mortality [1]. Despite its high mortality rate and high rates of ICU utilization, ARDS remains critically misdiagnosed and globally under-diagnosed in the ICU settings [1]. Furthermore, increasing ARDS severity is associated with increased mortality rate [6]. The PaO_2_/FiO_2_ ratio categorizes ARDS patients according to the severity of their oxygenation deficit without considering the level of applied PEEP in the assessment of lung severity. The PaO_2_/FiO_2_ ratio does not appropriately show the severity of ARDS for PEEP ≥ 5. However, the application of PEEP plays a significant role in improving oxygenation. It is well established that changes in PEEP alter the PaO_2_/FiO_2_ in lung-injured patients [29]. Attempting to predict lung severity and patient outcomes based solely in PaO_2_/FiO_2_ on this basis is inherent flawed. Thus, the stratification of ARDS patients as proposed by the Berlin criteria is useless for assessing severity of lung injury and could be of no benefit for enrolling patients into therapeutic clinical trials. The P/FP_E_ for PEEP ≥ 5 appropriately addressed Berlin’s definition gap in computing ARDS severity by including PEEP in the novel ratio. Clearly, our study showed that P/FP_E_ thresholds improved prediction of ARDS severity. This can lead to important medical implications by accurately anticipate specific treatment for each ARDS category, which could eventually decrease ARDS mortality. In other words, P/FP_E_ can represent a good solution for the clinical assessment of ARDS severity and as a guidance for treatment of ARDS.

Our study has several strengths. First, we have analyzed a large population of ARDS patients within their first three ICU days after onset. Second, we have described and validated our findings using both a large single-center database (MIMIC-III) and a large multicenter database (eICU). Third, we have investigated several ML predictive models for ARDS severity over time after ARDS onset. We believe that our approach is generalizable across other ARDS populations. However, we acknowledge some limitations to our study. First, our work is based on a retrospective analysis of data whose results concerning P/FP_E_ benefits should be confirmed in further prospective studies. Second, our analysis is concerned with the evolution and stratification of patients in their third ICU day after ARDS onset. Although the first 72-h are essential in the management and progression of ARDS patients, our study lacks the assessment of a long-term outcome (e.g., ICU mortality, 60-day mortality). Third, further longitudinal studies on complete evolution of ARDS patients could help to find out new evidence(s) on the management of ARDS since our ML results achieved outstanding improvements compared to the current state, with “fair” to “good” predictions of ARDS severity [42]. Forth, one could argue that extracorporeal membrane oxygenation (ECMO) is not considered in this study. ECMO is a clinical outcome and can only temporarily sustain severe ARDS patients to bridge periods of time when oxygenation through the lungs cannot be achieved via MV. Moreover, ECMO is a constrained resource that is not available in all ICUs. Hence, for the purpose of our study, we only considered patients receiving MV for > 48 h [32, 33]. Fifth, regarding the potential consequences of using the new ratio at the bedside, further studies are needed to examine whether it could help for clinical decision making and guiding therapy. Our study opens a possibility to better define ARDS severity, as a new research area for patient care improvement.

## Conclusions

This large study proposes a novel criterion based on the P/FP_E_ formula to assess ARDS severity using ML, which is significantly better than the current Berlin criteria using baseline PaO_2_/FiO_2_. Clinically, applying the proposed new criteria for ARDS severity enables clinical care physicians to assess lung severity by involving PEEP information. Moreover, being able to better adjust the severity profiles of ARDS patients will potentially improve the selection of more adequate therapeutic regimens for each ARDS category, which could contribute to reduce ARDS mortality. However, additional studies are required in order to confirm this. In both databases (MIMIC-III and eICU) and either in Berlin or P/FP_E_, scenario II (assessment of oxygenation deficit after 24 h of ARDS diagnosis and routine ICU treatment) was the best severity predictive scenario. From a ML perspective, P/FP_E_ outperformed PaO_2_/FiO_2_ in all ML models predicting ARDS severity after onset over time in all scenarios either in MIMIC-III or eICU. Accordingly, this study can serve as an example of how ML is a worth-considering technology to gain new insights in the development of ARDS predictive models which could contribute to improve ICU resource allocation and mortality reduction.

## Supplementary Information

**Additional file 1.** Predictors at 24-h and 48-h and other clinical outcomes, and their descriptive statistics in MIMIC-III and eICU.

## Data Availability

By request to M. Sayed and D. Riaño.

## Abbreviations

AECC: American–European Consensus Criteria
ARDS: Acute respiratory distress syndrome
AUC: Area under the receiver operating characteristic curve
CI: Confidence interval
CORR: Correlation between the predicted and actual values of the target variable
ECMO: Extracorporeal membrane oxygenation
eICU: EICU Collaborative Research Database
FiO_2_: Fraction of the oxygen in the inspired air
HR: Heart rate
LightGBM: Light Gradient Boosting Machine
ICU: Intensive care unit
ML: Machine learning
MIMIC-III: Medical Information Mart for Intensive Care Database
MV: Mechanical ventilation
PaCO_2_: Partial pressure of arterial CO_2_
PaO_2_: Arterial oxygen tension
PaO_2_/FiO_2_ or P/F: Ratio of partial pressure of arterial O_2_ to fraction of inspired O_2_
P/FP_E_: New ARDS severity criteria
PEEP or P_E_: Positive end-expiratory pressure
RF: Random forest
ROSE: Reevaluation of Systemic Early Neuromuscular Blockade
RR: Respiratory rate
SD: Standard deviation
SpO_2_: Oxygen saturation
XGBoost: EXtreme Gradient Boosting
