# Supplementary material for: Novel criteria to classify ARDS severity using a machine learning approach
Source: Crit Care. 2021 Apr 20;25:150. doi: 10.1186/s13054-021-03566-w (PMC8056190; doi:10.1186/s13054-021-03566-w)
Supplement: Supplementary file 1 — Additional file 1. Predictors at 24-h and 48-h and other clinical outcomes, and their descriptive statistics in MIMIC-III and eICU. [file 13054_2021_3566_MOESM1_ESM.docx]

***Supplementary Material***

**Novel criteria to classify ARDS severity using a machine learning approach**

**^a,*^ Mohammed Sayed,** M.Sc., **^a,*^ David Riaño,** PhD, **^b,c,d,*^ Jesús Villar**, MD, PhD

From

*(a) Banzai Research Group on Artificial Intelligence, Department of Computer Engineering, Universitat Rovira i Virgili, Av Paisos Catalans 26, 43007 Tarragona, Spain;*

(b) *Centro de Investigación Biomédica en Red de Enfermedades Respiratorias, Instituto de Salud Carlos III, Madrid, Spain;*

*(c) Multidisciplinary Organ Dysfunction Evaluation Research Network, Research Unit, Hospital Universitario Dr Negrín, Las Palmas de Gran Canaria, Spain;*

*(d) Keenan Research Center for Biomedical Science at the Li Ka Shing Knowledge Institute, St Michael's Hospital, Toronto, Ontario, Canada.*

(*) All the three authors are corresponding authors.

Address for correspondence (only for manuscript submission):

Dr. Jesús Villar. Research Unit, Hospital Universitario Dr. Negrín. Barranco de la Ballena s/n, 4^th^ floor -south wing. 35019 Las Palmas de Gran Canaria, Spain.

Phone: +34-928449413; Email: jesus.villar54@gmail.com

**SUPPLEMENTARY TABLES**

**Table S1. Input variables and their descriptive statistics in MIMIC-III at 24-h**

|  | **Mild** | **Moderate** | **Severe** | **All** |
| --- | --- | --- | --- | --- |
| *A. ARDS Patients* | ***669 (24.43%)*** | ***1,263(46.13%)*** | ***806 (29.44%)*** | ***2,738 (100%)*** |
| *B. Descriptive feature–* ***means and 95% CI*** | | | | |
| Age | 63.72 [62.52, 64.91] | 62.70 [61.82, 63.58] | 60.14 [58.97, 61.30] | 62.19 [61.59, 62.80] |
| PEEP | 5.87 [5.71, 6.02] | 6.95 [6.79, 7.12] | 9.57 [9.26, 9.87] | 7.46 [7.32, 7.59] |
| Heart Rate_Mean | 89 [87, 90] | 91 [90, 92] | 94 [93, 95] | 91 [91, 92] |
| Respiratory Rate_Mean | 19 [18, 19] | 20 [19, 20] | 22 [22, 23] | 20 [20, 21] |
| Heart Rate_Max | 110 [109, 112] | 113 [112, 114] | 117 [115, 118] | 114 [113, 114] |
| Heart Rate_Min | 73, [71, 74] | 74 [73, 75] | 77 [76, 78] | 75 [74, 75] |
| Respiratory Rate_Max | 27 [27, 28] | 29 [29, 30] | 32 [31, 32] | 29 [29, 30] |
| Respiratory Rate_Min | 12 [12, 13] | 13 [12, 13] | 14 [13, 14] | 13 [12, 13] |
| SpO_2__Mean | 98 [98, 99] | 97 [97, 98] | 96 [95, 96] | 97 [97, 98] |
| SpO_2__Max | 100 [99, 100] | 100 [99, 100] | 100 [99, 100] | 100 [99, 100] |
| SpO_2__Min | 92 [91, 93] | 90 [89, 90] | 86 [85, 87] | 89 [89, 90] |
|  |  |  |  |  |

**Table S2. Input variables and their descriptive statistics in MIMIC-III at 48-h**

|  | **Mild** | **Moderate** | **Severe** | **All** |
| --- | --- | --- | --- | --- |
| *A. ARDS Patients* | ***512 (33.71%)*** | ***778 (51.22%)*** | ***229 (15.08%)*** | ***1,519 (100%)*** |
| *B. Descriptive feature–* ***means and 95% CI*** | | | | |
| Age | 62.29 [60.89, 63.69] | 60.45 [59.28, 61.61] | 58.11 [55.92, 60.29] | 60.72 [59.89, 61.55] |
| PEEP | 7.32 [7.02, 7.62] | 9.33 [8.94, 9.72] | 11.91 [10.89, 12.94] | 9.04 [8.76, 9.32] |
| Heart Rate_Mean | 91 [89, 92] | 93 [92, 94] | 97 [95, 99] | 93 [92, 94] |
| Respiratory Rate_Mean | 20 [20, 21] | 21 [20, 21] | 23 [22, 23] | 21 [20, 21] |
| Heart Rate_Max | 113 [111, 115] | 115 [114, 117] | 121 [118, 124] | 116 [114, 117] |
| Heart Rate_Min | 74 [73, 76] | 76 [75, 78] | 78 [76, 80] | 76 [75, 77] |
| Respiratory Rate_Max | 29 [28, 30] | 30 [29, 31] | 33 [32, 34] | 30 [29, 31] |
| Respiratory Rate_Min | 13 [13, 14] | 13 [13, 14] | 14 [13, 14] | 13 [13, 14] |
| SpO_2__Mean | 98 [97, 98] | 97 [96, 97] | 96 [95, 96] | 97 [96, 97] |
| SpO_2__Max | 100 [99, 100] | 100 [99, 100] | 100 [99, 100] | 100 [99, 100] |
| SpO_2__Min | 90 [89, 91] | 88 [87, 89] | 85 [83, 86] | 88 [88, 89] |
|  |  |  |  |  |
|  | | | | |

**Table S3. Other clinical outcomes and their descriptive statistics in MIMIC-III at 24-h, 48-h, and 72-h**

|  | **Database** |  |  | | **ICU Day; (*N*)** | **Criteria** | **ARDS Class** | **ICU Mortality Rate (%)**  ***Mean and 95% CI*** | | **Duration of MV(hours)**  ***Mean and 95% CI*** | |
| --- | --- | --- | --- | --- | --- | --- | --- | --- | --- | --- | --- |
|  | ***Mimic III*** |  |  | | Day 1 (*669*) | ***Berlin definition*** | ***Mild*** | *24.07 [20.8, 27.3]* | | *184.18 [173.77, 194.59]* | |
|  |  |  |  | | Day 2 (*512*) |  |  | *23.44,[19.76, 27.12]* | | *192.58 [180.27, 204.90]* | |
|  |  |  |  | | Day 3 (*506*) |  |  | *23.72 [19.99, 27.44]* | | *192.07 [180.92, 203.23]* | |
|  |  |  |  | | Day 1 (*1,263*) |  | ***Moderate*** | *25.73 [23.3, 28.1]* | | *194.32 [186.37, 202.27]* | |
|  |  |  |  | | Day 2 (*778*) |  |  | *27.51 [24.36, 30.65]* | | *212.81 [201.97, 223.64]* | |
|  |  |  |  | | Day 3 (*678*) |  |  | *30.09 [26.63, 33.55]* | | *222.54 [210.67, 234.41]* | |
|  |  |  |  | | Day 1 (*806*) |  | ***Severe*** | *30.52 [27.3, 33.7]* | | *217.73 [206.92, 228.54]* | |
|  |  |  |  | | Day 2 (*229*) |  |  | *37.99 [31.66, 44.33]* | | *245.44 [224.27, 266.62]* | |
|  |  |  |  | | Day 3 (*157*) |  |  | *40.13 [32.38, 47.88]* | | *245.77 [216.74, 274.79]* | |
|  |  |  |  | | Day 1 (*554*) | ***New severity criteria*** | ***Mild*** | *25.5 [22, 29]* | | *183.51 [171.95, 195.07]* | |
|  |  |  |  | | Day 2 (*310*) |  |  | *26.5 [22, 31]* | | *177.17 [163.07, 191.28]* | |
|  |  |  |  | | Day 3 (*287*) |  |  | *25.1 [20, 30]* | | *182.83 [168.10, 197.55]* | |
|  |  |  |  | | Day 1 (*930*) |  | ***Moderate*** | *26.8 [24, 30]* | | *185.27 [176.91, 193.63]* | |
|  |  |  |  | | Day 2 (*482*) |  |  | *26.3 [22, 30]* | | *187.23 [175.88, 198.58]* | |
|  |  |  |  | | Day 3 (*453*) |  |  | *28.7 [25, 33]* | | *190.26 [178.40, 202.12]* | |
|  |  |  |  | | Day 1 (*1,254*) |  | ***Severe*** | *27.3 [25, 30]* | | *215.45 [206.60, 224.29]* | |
|  |  |  |  | | Day 2 (*727*) |  |  | *29.2 [26, 32]* | | *240.99 [228.54, 253.45]* | |
|  |  |  |  | | Day 3 (*601*) |  |  | *30.8 [27, 34]* | | *246.25 [232.55, 259.95]* | |
|  |  | | |  | | | | |  | |  |

**Table S4. Input variables and their descriptive statistics in eICU at 24-h**

|  | **Mild** | **Moderate** | **Severe** | **All** |
| --- | --- | --- | --- | --- |
| *A. ARDS Patients* | ***1,549 (30.06%)*** | ***2,361 (45.82%)*** | ***1,243 (24.12%)*** | ***5,153 (100%)*** |
| *B. Descriptive feature–* ***means and 95% CI*** | | | | |
| Age | 64.04 [63.28, 64.80] | 64.14 [63.56, 64.73] | 61.11 [60.27, 61.95] | 63.38 [62.97, 63.79] |
| PEEP | 5.72 [5.60, 5.83] | 6.25 [6.14, 6.35] | 8.55 [8.36, 8.75] | 6.64 [6.56, 6.73] |
| FiO_2_ | 0.51 [0.50, 0.52] | 0.59 [0.59, 0.61] | 0.85 [0.84, 0.86] | 0.63 [0.63, 0.64] |
| PaO_2_ | 128.05 [125.39, 130.72] | 98.84 [97.54, 100.14] | 84.19 [82.79, 85.59] | 104.09 [102.94, 105.24] |
| PaCO_2_ | 40.74 [40.19, 41.29] | 44.05 [43.52, 44.57] | 45.99 [45.35, 46.64] | 43.52 [43.19, 43.86] |
|  |  |  |  |  |
|  | | | | |

**Table S5. Input variables and their descriptive statistics in eICU at 48-h**

|  | **Mild** | **Moderate** | **Severe** | **All** |
| --- | --- | --- | --- | --- |
| *A. ARDS Patients* | ***1,098 (36.83%)*** | ***1,454 (48.78%)*** | ***429 (14.39%)*** | ***2,981 (100%)*** |
| *B. Descriptive feature–* ***means and 95% CI*** | | | | |
| Age | 64.73 [63.85, 65.61] | 63.29 [62.54, 64.06] | 59.97 [58.67, 61.28] | 63.35 [62.82, 63.88] |
| PEEP | 6.11 [5.95, 6.27] | 7.05 [6.91, 7.19] | 9.93 [9.55, 10.31] | 7.12 [7.00, 7.23] |
| FiO_2_ | 0.42 [0.41, 0.43] | 0.53 [0.52, 0.53] | 0.83 [0.81, 0.85] | 0.53 [0.52, 0.54] |
| PaO_2_ | 102.31 [100.52, 104.09] | 83.33 [82.23, 84.43] | 74.73 [72.82, 76.63] | 89.08 [88.12, 90.05] |
| PaCO_2_ | 39.46 [38.91, 40.01] | 41.89 [41.34, 42.44] | 44.02 [42.94, 45.09] | 41.30 [40.93, 41.68] |
|  |  |  |  |  |
|  | | | | |

**Table S6. Other clinical outcomes and their descriptive statistics in eICU at 24-h, 48-h, and 72-h**

|  | **Database** |  | **ICU Day; (*N*)** | **Criteria** | **ARDS Class** | **ICU Mortality Rate (%)**  ***Mean and 95% CI*** | **Duration of MV(days)**  ***Mean and 95% CI*** |
| --- | --- | --- | --- | --- | --- | --- | --- |
|  | ***eICU*** |  | Day 1 (*1,549*) | ***Berlin definition*** | ***Mild*** | *14.27 [12.52, 16.01]* | *6.28 [6.06, 6.49]* |
|  |  |  | Day 2 (*1,098*) |  |  | *16.21 [14.03, 18.39]* | *6.75 [6.47, 7.03]* |
|  |  |  | Day 3 (*872*) |  |  | *16.39 [13.94, 18.86]* | *7.58 [7.25, 7.91]* |
|  |  |  | Day 1 (*2,361*) |  | ***Moderate*** | *18.68 [17.11, 20.25]* | *6.98 [6.78, 7.19]* |
|  |  |  | Day 2 (*1,454*) |  |  | *23.52 [21.33, 25.70]* | *7.94 [7.65, 8.24]* |
|  |  |  | Day 3 (*1,025*) |  |  | *23.51 [20.91, 26.11]* | *8.75 [8.40, 9.10]* |
|  |  |  | Day 1 (*1,243*) |  | ***Severe*** | *28.72 [26.20, 31.24]* | *8.30 [7.96, 8.65]* |
|  |  |  | Day 2 (*429*) |  |  | *43.12 [38.42, 47.83]* | *9.09 [8.45, 9.72]* |
|  |  |  | Day 3 (*429*) |  |  | *43.12 [38.42, 47.83]* | *9.09 [8.45, 9.72]* |
|  |  |  | Day 1 (*1,309*) | ***New severity criteria*** | ***Mild*** | *14.36 [12.46, 16.27]* | *6.17 [5.94, 6.40]* |
|  |  |  | Day 2 (*849*) |  |  | *15.31 [12.89, 17.74]* | *6.19 [5.89, 6.49]* |
|  |  |  | Day 3 (*698*) |  |  | *14.61 [11.99, 17.24]* | *7.39 [7.04, 7.76]* |
|  |  |  | Day 1 (*2,053*) |  | ***Moderate*** | *18.11 [16.45, 19.79]* | *6.72 [6.51, 6.94]* |
|  |  |  | Day 2 (*1,182*) |  |  | *20.73 [18.41, 23.04]* | *7.64 [7.33, 7.95]* |
|  |  |  | Day 3 (*822*) |  |  | *20.80 [18.02, 23.58]* | *8.18 [7.82, 8.53]* |
|  |  |  | Day 1 (*1,791*) |  | ***Severe*** | *25.63 [23.60, 27.65]* | *8.18 [7.89, 8.47]* |
|  |  |  | Day 2 (*950*) |  |  | *34.74 [31.70, 37.77]* | *9.01 [8.60, 9.43]* |
|  |  |  | Day 3 (*806*) |  |  | *36.72 [33.39, 40.06]* | *9.42 [8.97, 9.87]* |
|  |  | | | | | |  |

**SUPPLEMENTARY FIGURES**

**Figure S1**

**Figure S2**

**Figure S3**

**Figure S4**
